# Cerner real-world data (CRWD) - A de-identified multicenter electronic health records database

**DOI:** 10.1016/j.dib.2022.108120

**Published:** 2022-03-31

**Authors:** Louis Ehwerhemuepha, Kimberly Carlson, Ryan Moog, Ben Bondurant, Cheryl Akridge, Tatiana Moreno, Gary Gasperino, William Feaster

**Affiliations:** a Children's Health of Orange County, Orange, CA 92868 USA; b Chapman University, Orange, CA 92866 USA; c Cerner Corporation, Kansas City, MO USA

**Keywords:** Cerner Real-World Data^TM^(CRWD), COVID-19, SARS-CoV-2, Electronic Health Records (EHR), HealtheIntent, HealtheDataLab™, Cerner learning Health Network^SM^ (LHN)

## Abstract

*Cerner Real-World Data*^TM^ (CRWD) is a de-identified big data source of multicenter electronic health records. Cerner Corporation secured appropriate data use agreements and permissions from more than 100 health systems in the United States contributing to the database as of March 2022. A subset of the database was extracted to include data from only patients with SARS-CoV-2 infections and is referred to as the Cerner COVID-19 Dataset. The December 2021 version of CRWD consists of 100 million patients and 1.5 billion encounters across all care settings. There are 2.3 billion, 2.9 billion, 486 million, and 11.5 billion records in the condition, medication, procedure, and lab (laboratory test) tables respectively. The 2021 Q3 COVID-19 Dataset consists of 130.1 million encounters from 3.8 million patients. The size and longitudinal nature of CRWD can be leveraged for advanced analytics and artificial intelligence in medical research across all specialties and is a rich source of novel discoveries on a wide range of conditions including but not limited to COVID-19.

## Specifications Table

**Table:** 

Subject	Big Data Analytics
Specific subject area	Multicenter electronic health records database
Type of data	Electronic health records
How data were acquired	Data use agreements and permissions from individual health systems were obtained from clients of Cerner across the United States. Data from each health system were combined and de-identified into a single database.
Data format	Parquet Tables
Parameters for data collection	Electronic health records from each health system that fits into a Structured Query Language tabular format excluding most freetext entries, clinical notes, and images.
Description of data collection	To create CRWD, each contributor's HealtheIntent data (copy of the EHR) is retrieved for processing and merged into a data warehouse which is then processed to help reduce duplication of identifiers between contributors. After de-duplication, the data is deidentified on an individual patient level by removing fields that contain personal identifiable information (PII) and date-shifting all date/timestamp values. Unique identifiers masking the health systems was created in addition to corresponding U.S. census regions.
Data source location	Cerner Corporation North Kansas City, MO USA
Data accessibility	Readers may request access to Cerner Real World Data by (1) licensing the database for a research project that is granted approval by the Cerner Learning Health Network Governance Council. (2) Access is also available to organizations who are contributing data to CRWD. For inquiries about CRWD including information on data use agreements reach out to realworlddata@cerner.com while inquiries about the COVID-19 dataset can be sent to COVIDDataLab@cerner.com.

## Value of the Data

• Cerner Real-World Data^TM^ (CRWD) is designed to help users answer deep and complex research questions using data from multiple health systems and heterogenous patient groups. It reduces bias in research due to data from homogenous population that may be inherent in single center studies, and it provides larger sample sizes for rare disease studies. It also ensures that most conditions can be modeled using machine learning given the larger sample sizes.
• All researchers, including academic, health system, or life sciences investigators can access CRWD if their healthcare organization is contributing de-identified data to the dataset or by contracting with Cerner through a Learning Health Network (LHN) for access to HealtheDataLab (a cloud-parallel distributed learning framework) to conduct an approved research project. Other interested researchers or organizations can apply for access to CWRD pending approval.
• With this longitudinal database, researchers can analyze detailed sets of deidentified clinical data at the patient level and develop statistical and machine learning models that may be implementable in various healthcare settings.

## 1 Data Description

**Fig. 1:**
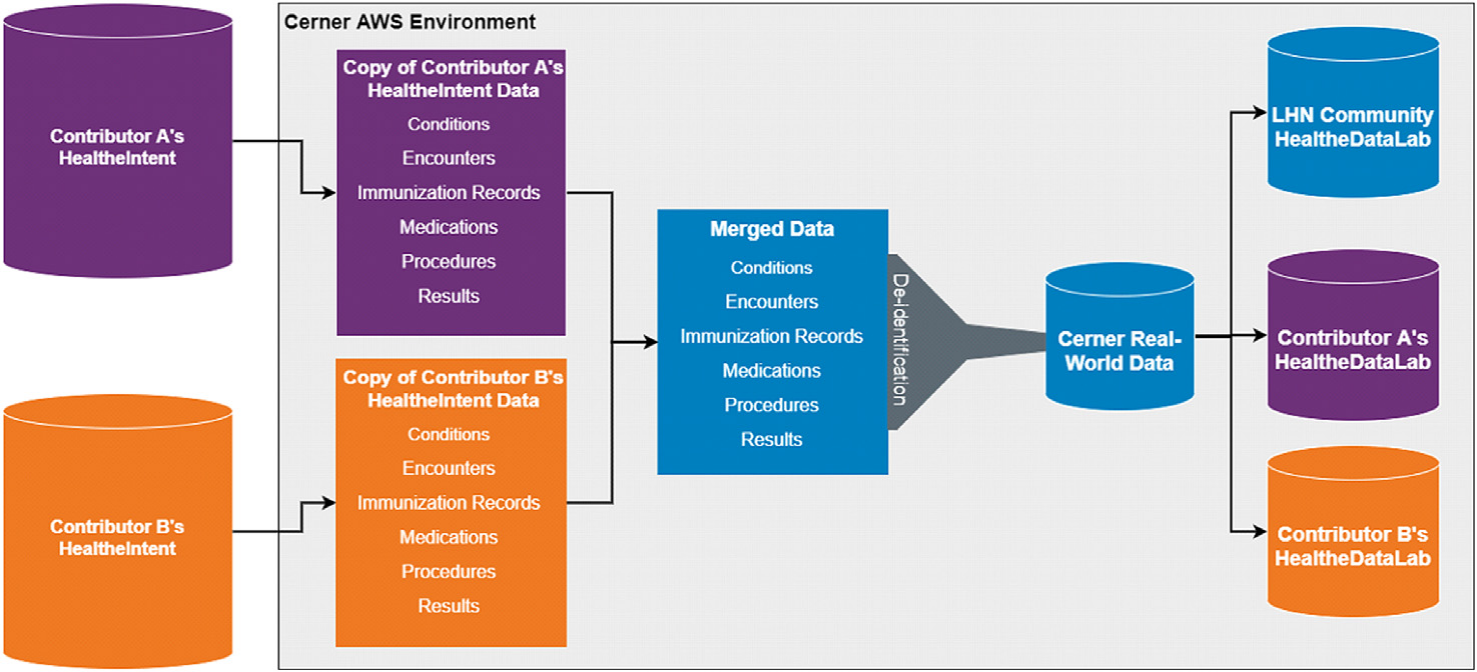
Compilation of the CRWD database.

Fig. 1 visually describes the compilation of CRWD. The tables in CRWD include encounters, demographics, conditions, immunizations, medications, medication administrations, order lists, procedures, and results. The encounter table consists of pertinent information regarding patients’ episode of care (or encounter with the health system). It is comprised of data on the encounter class (inpatient, outpatient, emergency department, etc.), datetime fields (service date, hospitalization date, discharge date, etc.), insurance presented during the visit (Medicare, Medicaid, Commercial, etc.), unique identifiers for the patient, encounter, and the health system visited among others. The demographics table includes data on birth sex, gender, date of birth, race, ethnicity, tenant (health system), and one-digit zip code for tenant. Information on the diagnoses of patients are captured within the conditions table including information on diagnosis rank, condition coding system identifiers (International Classification of Diseases, Tenth Revision, Clinical Modification (ICD-10-CM), International Classification of Diseases, Ninth Revision, Clinical Modification (ICD-9-CM), Systemized Nomenclature of Medicine – Clinical Terms (SNOMED), etc.), and the class of condition (admitting, working, discharge, final, etc.). The labs, measurements, and clinical events tables keep track of clinical events data including vital signs, results of clinical assessments, and laboratory test result. Data on immunization, medication orders, and procedures are captured within the corresponding tables and include full information required for research with the data.

**Fig. 2:**
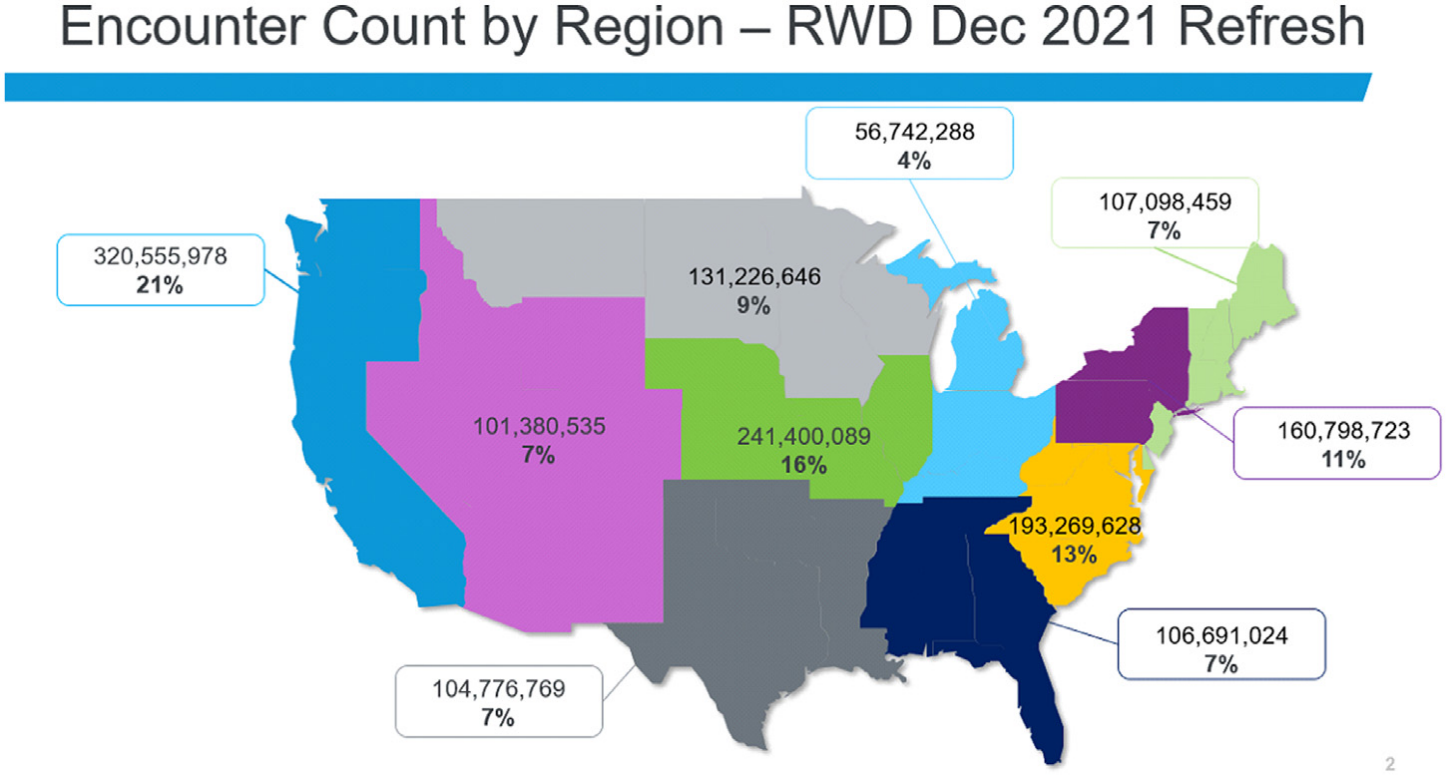
Geographical distribution of the CRWD, encounters per U.S. region, December 2021.

The 2021 Q3 COVID-19 Dataset is a subset of CRWD, and Table 1 identifies the eight core data tables included in the COVID-19 database and which CRWD tables were used to populate it. The December 2021 version of the CRWD consists of data from 117 health systems across the United States. It includes data from 100 million patients and more than 1.5 billion encounters across all care settings. A geographical distribution of encounters is shown in Figure 2. An overview of the contents (table, item) and size (numbers of patients and encounters) of the database tables in CRWD are listed in Table 2. Counts are calculated using distinct person IDs which leverage a multipoint match algorithm to account for and remove duplicates within a single health system; patients who have visited multiple health systems may appear more than once in the data.

**Table 1:** Correspondence between CRWD and the COVID-19 database.

COVID-19 Database Table	Primary CRWD Source Table(s)
allergy	allergy
allergy_reaction	allergy
clinical_event	clinical_event
condition	condition
covid_labs	lab
demographics	demographics
encounter	encounter, demographics
immunization	immunization
lab	lab
measurement	measurement
med_rec_compliance	medication, order_list
medication	medication
procedure	procedure

**Table 2:** Description of contents of the December 2021 version of CRWD.

Table name	Item	Number of Patients	Number of Encounters
Encounter	Pediatric inpatient	7,249,035	10,882,399
Encounter	Pediatric emergency department	11,722,447	34,914,405
Encounter	Pediatric outpatient	24,266,779	166,957,515

Encounter	Adult inpatient	15,871,479	37,380,794
Encounter	Adult emergency department	24,136,017	78,002,292
Encounter	Adult outpatient	48,242,055	669,703,048

Condition	Infectious and parasitic diseases (A00-B99)	8,900,949	19,585,205
Condition	Neoplasms (C00-D49)	5,442,981	31,002,741
Condition	Disease of the blood and blood-forming organs and certain disorders involving the immune mechanism (D50-D89)	6,307,437	23,103,433
Condition	Endocrine, nutritional and metabolic diseases (E00-E89)	16,819,011	105,037,877
Condition	Mental, behavioral and neurodevelopmental disorders (F01-F99)	13,530,099	60,514,605
Condition	Disease of the nervous system (G00-G99)	10,672,036	41,488,133
Condition	Disease of the eye and adnexa (H00-H59)	5,166,226	11,306,402
Condition	Diseases of the ear and mastoid process (H60-H95)	6,348,385	14,440,841
Condition	Disease of the circulatory system (I00-I99)	14,044,208	97,464,096
Condition	Diseases of the respiratory system (J00-J99)	18,461,638	65,375,969
Condition	Diseases of the digestive system (K00-K95)	15,426,814	52,007,729
Condition	Disease of the skin and subcutaneous tissue (L00-L99)	9,692,694	25,352,196
Condition	Diseases of the musculoskeletal system and connective tissue (M00-M99)	19,347,816	88,953,759
Condition	Disease of the genitourinary system (N00-N99)	14,492,187	54,591,100
Condition	Pregnancy, childbirth, and puerperium (O00-O9A)	2,869,918	12,448,034
Condition	Certain conditions originating in the perinatal period (P00-P96)	2,068,973	3,757,005
Condition	Congenital malformations, deformations and chromosomal abnormalities (Q00-Q99)	2,537,097	8,563,309
Condition	Symptoms, signs and abnormal clinical and laboratory findings, not elsewhere classified (R00-R99)	34,355,822	158,411,418
Condition	Injury, poisoning and certain other consequences of external causes (S00-T88)	18,378,852	45,720,755
Condition	Codes for special purposes (U00-U85)	1,626,660	2,635,199
Condition	External causes of morbidity (V00-Y99)	12,592,946	24,843,751
Condition	Factors influencing health status and contact with health services (Z00-Z99)	36,522,771	218,705,416

Allergy	Allergies reported during encounter	52,068,306	70,517,620
Clinical event	Clinical events excluding vital signs and laboratory test results	55,239,445	374,382,082
Demographics	Patient demographic information	100,869,790	NA
Immunization	Patient immunization records	17,465,249	44,014,386
Lab	Information on laboratory tests performed and corresponding results	51,846,090	298,532,541
Measurement	Data on vital signs, height, and weight	56,164,999	374,432,541
Medication	Medications that were ordered or prescribed	53,712,382	338,169,057
Medication administration	Information of medication administration	26,609,473	78,117,057
Order list	Orders not captured in medications or results table	36,387,299	205,471,562
Procedure	This contains information on procedures including surgical encounters	37,272,972	171,540,666

**Fig. 3:**
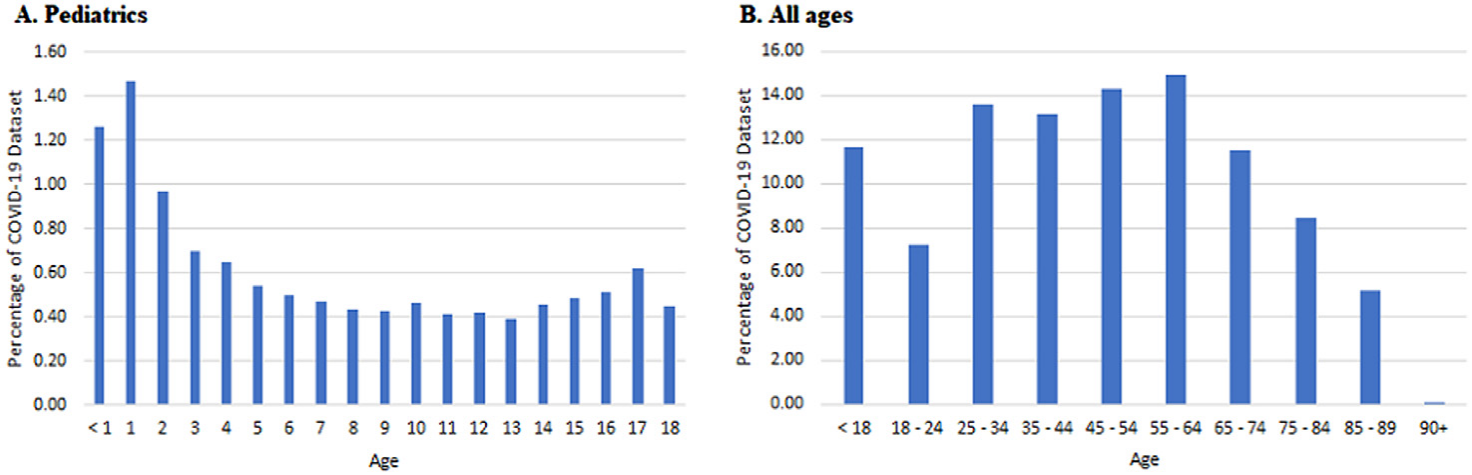
Age distribution of the COVID-19 data set.

There is usually a 4-month lag between the release of CRWD and release of corresponding COVID-19 data set. As a result, the version of the COVID-19 dataset available at the time of writing was the 2021 third quarter version. The 2021 Q3 COVID-19 Dataset consists of 3.8 million patients and 130.1 million encounters from 110 health systems in the United States. Among these, 2.1 million patients had 2.7 million inpatient or emergency department encounters with infections with SARS-CoV-2 virus. Additional de-identification processes resulted in combining pediatric patients that are 17 years or younger into a single age group occluding the pediatric distribution of patients. Both the pediatric and adult age distribution of the COVID-19 data set are shown in Fig. 3. Description of the contents (table, item) and size (numbers of patients and encounters) of the database tables in the COVID-19 data set is shown in Table 3.

**Table 3:** Description of contents of the 2021 Q3 COVID-19 Dataset.

Table name	Item	Number of Patients	Number of Encounters
Encounter	Pediatric inpatient	247,597	498,717
Encounter	Pediatric emergency department	555,746	2,025,078

Encounter	Adult inpatient	1,942,636	6,968,315
Encounter	Adult emergency department	2,228,542	10,853,010

Allergy	Allergy records	3,160,474	4,913,834
Allergy reaction	Allergy reaction details	414,853	742,621
Clinical event	Clinical events excluding vital signs and laboratory test results	3,784,204	43,132,105
Condition*	Patient diagnoses and conditions	3,787,176	66,295,823
COVID labs	Information on COVID-19 laboratory tests	3,468,290	5,836,584
Demographics	Patient demographic information	3,836,912	NA
Immunization	Patient immunization records	1,153,660	2,833,849
Labs	Information on laboratory tests	3,790,477	33,805,117
Measurement	Data on vital signs, height, and weight	3,783,959	41,457,721
Medication*	Medications that were ordered or prescribed	3,605,825	36,782,389
Medication reconciliation and compliance*	Medication reconciliation and compliance status	2,913,450	20,506,531
Procedure*	Information on procedures including surgeries	2,713,877	16,400,517

⁎ Includes data on patients found or suspected to be infected with SARS-CoV-2 including their historical medical records from 2015

## 2 Experimental Design, Materials and Methods

The development of CRWD was initiated in 2019. As of December 2021, 117 health systems in the United States (U.S.) have formally agreed to contribute deidentified patient data to the database in exchange for benefits which include access to the entire multicenter database. The number of participating health systems is expected to grow.

CRWD has its roots in *HealtheIntent* [1,^2^,3], a Cerner EHR-agnostic population health management platform that aggregates and standardizes all clinical data sources at each health system regardless of EHR vendor. To create CRWD, each contributor's *HealtheIntent^SM^* data is transferred into the Cerner Amazon Web Services (AWS) environment for processing. Data from each contributor is merged into an aggregated data warehouse which is then processed to help reduce duplication of identifiers (e.g. person IDs or encounter IDs) between contributors. After de-duplication, the data is de-identified on an individual patient level by removing fields that contain personal identifiable information (PII) (e.g. first name, last name, address, phone number, unstructured information) as well as date-shifting all date values. Additional details on the U.S. policy on deidentification can be found on the US Department of Health and Human Services website [4]. Date shifting is done on a patient level by assigning a patient a random date shift value that is a multiple of ±7. Each patient's dates are then shifted by this value, preserving the day of the week for all data captured on the same patient as well as the temporal relationship between clinical events. Data is also de-identified on the health system level by removing fields which contain health system-identifying information such as the name or address of the contributing health system. Additional details on the U.S. policy on deidentification can be found on the US Department of Health and Human Services website [4].

With this longitudinal database, researchers can analyze detailed sets of de-identified clinical data at the patient level. Researchers can access CRWD if their healthcare organization is contributing de-identified data to the dataset or by contracting with Cerner through a Learning Health Network (LHN) for access to conduct an approved research project. Currently, access is limited to U.S. researchers only; however, CRWD may be available for select international researchers in the future. Interested researchers or organizations can apply for access to CWRD, pending approval of proposed research by a data governance council within the LHN and appropriate data use agreements.

All researchers, including academic, health system, or life sciences investigators, who wish to gain access to CRWD must submit a standard data access proposal to Cerner via the LHN (which is described in full details in the next subsection). The proposal provides information about study objectives, study populations, data elements and outcomes of interest, methods, and ultimate use of the analysis. Data access proposals are blinded of all identifying information before review and decision on approval by a data governance council. The LHN governance council is comprised of representatives from many contributing health systems, academic researchers, and a privacy domain expert. This governance council review data access proposals of all researchers regardless of LHN membership.

For all CRWD access, a data use agreement is required which governs the rules of interacting with the data. Rules include not exporting or downloading the data from the secure cloud-based data ecosystem nor attempting to re-identify any data. Analysis of CRWD is conducted in *HealtheDataLab*™ [1], the Cerner cloud-based data science ecosystem for analyzing data and predictive model development. *HealtheDataLab* is built and deployed by AWS and is designed to help users answer deep and complex research questions using statistical and data-science oriented tools that query data, extract and transform datasets into research-ready formats, build complex models and algorithms and validate findings. Available open-source tools in *HealtheDataLab* include Apache Spark™, Jupyter™, Python®, Spark R, and Spark SQL.

Researchers from non-contributing health systems, universities, or organizations can apply for access to CRWD to conduct research on approved research projects. Access and work with the CRWD are not without financial costs due to the size of the database and the state-of-the-art cloud computing resources required for hosting and managing it. These researchers (from non-contributing health systems) can gain access to CRWD via contractual agreement with the LHN covering both the need to guard and protect the privacy of contributing health systems and to cover the cost of data access and preprocessing using cloud computing.

## 2 The Learning Health Network (LHN)

The *Cerner Learning Health Network*^SM^ (LHN) is a collaboration of healthcare organizations that leverage EHR data for research and to improve clinical care. As of March 2022, 81 U.S. health systems in 39 states and the District of Columbia have joined the LHN, and they comprise of over 45,000 hospital beds and more than 2,222 facilities. Member organizations agree for Cerner to map their institutions’ de-identified, site-anonymized patient data for use in approved research. In return, contributing organizations receive benefits including complimentary access to CRWD and HealtheDataLab, as well as opportunities to participate in a variety of federal and industry-sponsored research studies, share learnings and collaborate with other members in the network, and propose their own research ideas. Any healthcare organization, academic, provider-focused, rural, or community-based health systems, can opt-in to join the LHN by signing a data network agreement to contribute their de-identified patient data to CRWD.

While data sharing is inherent to the LHN, a key focus is on operationalizing research tools to support clinical studies and create opportunities for members to participate in them. Study tools include patient recruitment, data capture and quality, chart review, patient adherence, and risk calculation. Designed to be a continual data quality improvement loop, the LHN pushes further cleansed data back into the network for members to leverage for research purposes.

## 3 Curating the CRWD COVID-19 De-identified Database

The COVID-19 pandemic presented an immediate need for data that could be used for research of risk factors, conditions, outcomes, and potential therapies. The Cerner COVID-19 database, which launched in April 2020, is a curated data set of patients with possible SARS-CoV-2 infection created from de-identified data obtained from CRWD. To qualify a patient for inclusion, an encounter must have a service date of December 1, 2019 or later; an encounter type of emergency, inpatient, admitted for observation, inpatient hospice care, or urgent care encounter; and either a diagnosis related COVID-19 disease from the CRWD condition table or a positive result from a qualifying laboratory code (CRWD result table). In summary, patients qualified for the database with a COVID infection code or COVID exposure code, and patients with a negative COVID-19 test qualified for the cohort if the test was completed on one of the qualifying encounters.

Although there is a direct correlation between data tables across the two databases, the structure of the COVID-19 database has been simplified to facilitate more efficient analysis, which aims to help reduce burden on end users. For example, some CRWD tables include data that were nested within complex data structures such as structs and arrays that are not intuitive for new users of Apache Spark. In preparing the COVID-19 data model, the nested data structures were re-engineered to accommodate users that are accustomed to working with flat files and basic Structured Query Language (SQL) tabular data. The steps taken to derive the COVID-19 database include the following:

Identify all encounters that would qualify a patient for inclusion in the database using confirmed laboratory findings for SARS-CoV-2 and diagnosis codes corresponding to COVID-19.

For each person in the cohort, obtain data from all CRWD encounters, conditions, and medications having a service date on or after 1/1/2015. Calculate derived variables (e.g., age at time of encounter) and transform the data to fit the COVID-19 data model and add metadata. Examples of metadata elements include binary indicators to help identify qualifying encounters and various record counts, which are intended to help offer insight into the availability of data for each patient and assist with the selection of study cohorts.

Variability in the way health systems report qualitative lab results has led to the need for some standardization. This standardization is reflected in the “covid_labs” table, which is unique to the COVID-19 database but uses the CRWD result table as its source. The mapping done here evaluates both text and codified results associated with qualifying lab codes and translates each entry into one of five standard categories: Positive, Negative, Indeterminate, Not done, or Unknown. Some examples of values that are mapped include POS, POSITIVE, Neg., NEG, ND, INDET, and Unk.

Once all encounters that would qualify a patient have been identified, a table is created that includes the list of the unique person ID values represented. This table is the cohort and serves as a list that can be joined to the CRWD tables to extract the full range of data to be included in the COVID-19 database.

## Ethics Statement

The EHR data collected was part of routine treatment and not originally collected for research. It is fully de-identified and therefore does not require patient consent. Furthermore, there are more than 100 million patients in the database making consent impractical. Research has been carried out in accordance with The Code of Ethics of the World Medical Association (Declaration of Helsinki). Conducting research with de-identified data does not meet regulatory criteria for research involving human subjects research, therefore human subjects research regulations regarding informed consent does not apply. All HIPPA guidelines were followed.

## CRediT Author Statements

**Louis Ehwerhemuepha:** All authors contributed to the design, acquisition of data, interpretation of data/results, drafting and revising the article critically for important intellectual content and approved the manuscript. Louis Ehwerhemuepha conceived of the study and led it. **Kimberly Carlson:** All authors contributed to the design, acquisition of data, interpretation of data/results, drafting and revising the article critically for important intellectual content and approved the manuscript. **Ryan Moog:** All authors contributed to the design, acquisition of data, interpretation of data/results, drafting and revising the article critically for important intellectual content and approved the manuscript. **Ben Bondurant:** All authors contributed to the design, acquisition of data, interpretation of data/results, drafting and revising the article critically for important intellectual content and approved the manuscript. **Cheryl Akridge:** All authors contributed to the design, acquisition of data, interpretation of data/results, drafting and revising the article critically for important intellectual content and approved the manuscript. **Tatiana Moreno:** All authors contributed to the design, acquisition of data, interpretation of data/results, drafting and revising the article critically for important intellectual content and approved the manuscript. **Gary Gasperino:** All authors contributed to the design, acquisition of data, interpretation of data/results, drafting and revising the article critically for important intellectual content and approved the manuscript. **William Feaster:** All authors contributed to the design, acquisition of data, interpretation of data/results, drafting and revising the article critically for important intellectual content and approved the manuscript.

## Declaration of Competing Interest

The authors declare no conflict of interest for this article.

## Data Availability

Cerner Real-World Data (Original data).
